# Congested Crowd Counting via Adaptive Multi-Scale Context Learning †

**DOI:** 10.3390/s21113777

**Published:** 2021-05-29

**Authors:** Yani Zhang, Huailin Zhao, Zuodong Duan, Liangjun Huang, Jiahao Deng, Qing Zhang

**Affiliations:** 1 School of Computer Science and Information Engineering, Shanghai Institute of Technology, Shanghai 201418, China; freezhangyani@gmail.com (Y.Z.); zhangqing0329@gmail.com (Q.Z.); 2 School of Electrical and Electronic Engineering, Shanghai Institute of Technology, Shanghai 201418, China; zhao_huailin@yahoo.com; 3 School of Mechatronical Engineering, Beijing Institute of Technology, Beijing 100081, China; zduan@bit.edu.cn (Z.D.); bitdjh@bit.edu.cn (J.D.)

**Keywords:** crowd counting, crowd density estimation, multi-scale context learning, crowd localization, remote sensing object counting

## Abstract

In this paper, we propose a novel congested crowd counting network for crowd density estimation, i.e., the Adaptive Multi-scale Context Aggregation Network (MSCANet). MSCANet efficiently leverages the spatial context information to accomplish crowd density estimation in a complicated crowd scene. To achieve this, a multi-scale context learning block, called the Multi-scale Context Aggregation module (MSCA), is proposed to first extract different scale information and then adaptively aggregate it to capture the full scale of the crowd. Employing multiple MSCAs in a cascaded manner, the MSCANet can deeply utilize the spatial context information and modulate preliminary features into more distinguishing and scale-sensitive features, which are finally applied to a 1 × 1 convolution operation to obtain the crowd density results. Extensive experiments on three challenging crowd counting benchmarks showed that our model yielded compelling performance against the other state-of-the-art methods. To thoroughly prove the generality of MSCANet, we extend our method to two relevant tasks: crowd localization and remote sensing object counting. The extension experiment results also confirmed the effectiveness of MSCANet.

## 1. Introduction

Crowd counting is an indispensable component for smart crowd analysis, to count the number of people and describe the crowd distribution. It plays a critical role in many areas, such as video surveillance [1], public security [2], human behavior analysis [3,4], and smart cities [5,6,7]. However, due to the frequent occurrence of scale variations and severe occlusions, in addition to the diverse crowd distributions, the task often faces great difficulties to accurately describe the crowd, especially in scenes of overcrowding.

**Figure 1:**
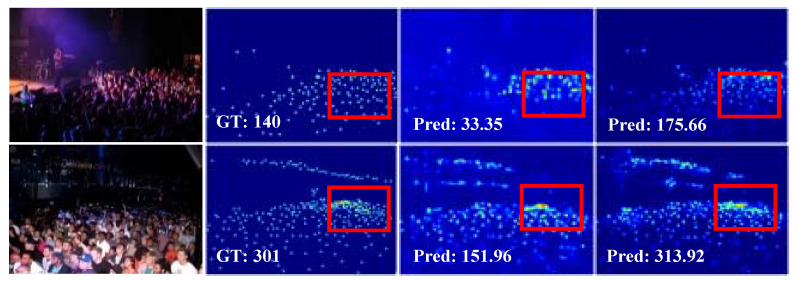
Representative examples in the UCF-QNRF dataset [17]. From left to right: input images, ground-truth, results of CSRNet [8], and the results of MSCANet. Compared to CSRNet, MSCANet can effectively handle the ambiguity of appearance between crowd and background objects.

Deep-learning-based methods have been the main method for solving this problem and have achieved quite a few significant improvements. However, challenges remain to be settled. For one thing, the results of crowd counting are not sufficiently accurate in severe occlusions, scale variations, and diverse crowd distribution scenes, especially under the circumstances of crowds that visually share a high similarity with their surroundings, as illustrated in the first column of Figure 1.

One of the major causes is that few studies have focused on the leveraging of spatial context representation. For instance, single-scale crowd counting networks [8,9] only employ convolution operations with a fixed kernel size, which may hurt the performance when the scale of the crowd changes. Multi-scale crowd counting networks [10,11,12,13,14] are carefully elaborate in order to portray different scales of people. They are still limited by the local receptive field of convolutional operation, and the features of the global spatial context cannot be fully utilized. Other studies [15,16] applied various modules to model scale-aware spatial context information; however, they merely aggregate different context features without any auxiliary processing, which cannot access the discriminative features and vastly harm the performance of the counting network.

Multi-scale context aggregation still has some space for improvement since only the typical features from a specific scale contribute to final crowd counting. We argue that the spatial context information of different scales should be aggregated in an adaptive way. For another, the estimated density maps are not reliable when considering the exact position even though the final reported count is precise. Unfortunately, in a majority of existing methods, precise crowd localization is rarely involved. Although, it is as significant as crowd counting since they are all fundamental tasks for crowd analysis.

Therefore, in this work, we propose a novel Adaptive Multi-scale Context learning mechanism for congested crowd counting and localization simultaneously, namely the Adaptive Multi-scale Context Aggregation Network (MSCANet). The kernel of the network is a Multi-scale Context Aggregation module (MSCA), which learns a multi-scale context representation in an adaptive way. MSCA introduces a multi-branch structure applying atrous convolution layers with different dilation rates aiming to encode multi-scale context features.

Then, the encoded features of the whole branches are aggregated layer by layer via a channel attention mechanism [18] to obtain a richer global scene representation. Multiple MSCAs concatenated in a cascaded manner are embedded in the MSCANet, where the subsequent up-sampling layer transforms the multi-scale features at each MSCA into higher-resolution representations. The high-level features from the last MSCA are further learned by a 1 × 1 convolution layer to output the two-channel results, including the crowd density map and crowd localization map.

MSCANet can be easily applied for various network backbones and learned in an end-to-end manner. Extensive experiments on three challenging public benchmarks (i.e., ShanghaiTech_Part_A, UCF_CC_50, and UCF-QNRF) showed that our model achieved compelling performance against the state-of-the-art methods. Additionally, to evaluate the generalization ability of our method, we extend MSCANet to two relevant tasks, i.e., crowd localization and remote sensing object counting. Our model was proven to generalize well and achieved superior localization results on the UCF-QNRF dataset and promising counting results on the RSOC dataset.

In summary, the main contributions of this paper are two-fold:

- We propose a MSCA to adaptively aggregate small-scale context representation with large-scale context representation in a cascade manner, which encodes more compact global context features for crowds at various scales.
- Employing multiple MSCAs, we introduce the MSCANet to obtain multi-scale context features with different resolutions. This can efficiently address the ambiguous appearance challenge, especially under crowded scenes with complex backgrounds.

The remainder of this paper is organized as follows. Section 2 reviews related work regarding crowd counting and crowd localization. Section 3 presents the proposed method for crowd counting and localization. Section 4 introduces the experiment settings and presents extensive experiment results. In Section 5, we conclude this paper and with some future directions.

This paper is built on our conference paper [19], and the content is extended from three aspects: First, we give a comprehensive review about crowd counting, crowd localization, and remote sensing object counting. Secondly, to evaluate the effectiveness of our MSCANet, we also conduct a crowd localization experiment on the UCF-QNRF dataset. Our qualitative and quantitative results demonstrate the superiority of our method. Thirdly, we extend our MSCANet to remote sensing object counting tasks and conduct extensive experiments on RSOC. Our method achieves promising results compared with other state-of-the-art methods.

## 2. Related Works

In this section, we will review some related works regarding crowd counting, crowd localization, and remote sensing object counting.

### 2.1. Crowd Counting

The task of crowd counting has been studied for many years. Research of crowd counting can be categorized as either detection-based methods or regression-based methods. Detection-based methods usually employ pedestrian or face detectors to recognize and localize crowds. However, the performance of the detectors deteriorates in congested crowd scenes due to occlusions and large-scale variations of the crowd. Regression-based methods establish the correspondences between the input image and the number of people. Conventional methods [20,21,22,23,24] use carefully designed handcrafted features and apply different regression methods to regress the final count number. Although they achieved progress, their performances are constrained due to the handcrafted features of their methods, which heavily rely on the specific crowd scenes.

Recently, with the renaissance of deep learning, many CNN-based crowd counting networks have been proposed, which cast the crowd counting problem as a crowd density estimation task. The research of CNN-based crowd counting methods is primarily three-fold: the design of the network architecture, the generation of the crowd density map, and the network optimization function. We will review the related work from the above three aspects as follows.

**Network structure**. Scale variation of the crowd head is a classical challenging problem of accurate crowd counting. Many counting networks [25,26,27,28,29,30,31,32,33] have been carefully designed to extract multi-scale features for handling this challenge. Early crowd counting networks typically employed multi-column structures [10,11,14,16,34] to model different scales of crowds. More recently, a graph network [35] was introduced to enhance scale-aware features. Perspective information of crowd scenes was also employed for networks [36,37] for improving the final counting performance. Later, research efforts were devoted to utilize context information efficiently.

For example, [38,39,40] proposed a crowd density classifier to provide each input image with a density-level label. The authors in [8] employed dilated convolutional layers to enlarge the receptive field of the network for extracting context information. Other researchers  [15] used spatial pyramid pooling [41] to enhance the different scales of context features for crowd counting. Benefiting from the efficiency of visual attention mechanisms for context information extracting [42,43,44,45], many attention-based counting networks [46,47,48,49] were designed and perform well on complicated crowd scenes in which the background objects have a similar appearance with foreground crowd.

Furthermore, to alleviate the effects of background objects for foreground crowd counting, foreground mask-based crowd counting networks [50,51,52,53] have been designed. Although the above methods achieved promising results, they rely on training data, and therefore their generalization ability is limited to new scenarios. Thus, some unsupervised domain-adaption methods [54,55] were developed for crowd counting and achieved satisfactory results.

**Crowd density map generation**. The density functions are considered as real-valued functions over pixel grids [56], whose integrals over image regions should match the object counts. Most CNN-based counting networks [9] applied a normalized 2-D Gaussian kernel to convolve with the head location for generating the crowd density map. Although they have achieved great performance, the density map generated by the normalized Gaussian kernel does not consider perspective changes, and thus cannot correctly model the crowd distribution, which hampers the performance of counting networks.

To solve this problem, Zhang et al. [10] employed geometry-adaptive kernels to solve the effects of perspective. Wan et al. [57] proposed a generation network to output the crowd density maps, which the counting network aims to optimize, and the counting network and generation network were trained end to end together. A. Sindagi et al. applied residual learning in a progressive fashion [58] to generate high-quality crowd density maps, and employed the MRF framework [59] to generate scale-aware density maps.

**Optimization function**. L2 loss was commonly used as the loss function in the CNN-based crowd counting method. However, its average effect led to blurry estimation and reduced the quality of the density map. Wan et al. [60] argued that the point annotations in the available crowd counting datasets could be considered as weak labels for density map estimation and proposed the Bayesian Loss, which constructs a density contribution probability model from the point annotations.

Adversarial Loss [61,62] was involved a Generator G and Discriminator D playing a two-player minimax game: G was trained to generate images to fool D while D was trained to distinguish synthetic images from the ground truth. It could avoid blur as well as incentivize sharp images since blurry outputs appear as unrealistic. Composition loss [17] was used for training and estimation of the three interrelated problems of counting, density map estimation, and localization, simultaneously. As a result, density maps can be “sharpened” until they approximate the localization map, whose integral should equal the true count. Cheng et al. [63] proposed a Maximum Excess over Pixels loss to learn spatial-aware crowd features.

### 2.2. Crowd Localization

Different from crowd counting, the task of crowd localization aims to acquire the exact locations of people in the image. It is also very challenging because people are very close to each other in the congested crowd scene. The methods of crowd localization can be divided into three categories: anchor-based localization methods, point-based localization methods, and heuristic-based localization methods.

**Anchor-based Localization Methods**. The anchor-based crowd localization methods draws on object detection, which designs a model to regress to the anchor box laid out by each person in advance. For instance, Liu et al. [64] proposed a DetNet based on Faster R-CNN [65] to detect sparse crowds. Lin et al. [66] employed the crowd density maps and scene depth maps to improve the detection performance of RetinaNet [67] for crowds. He et al. [68] utilized YoloV3 to detect crowds in the nearby region.

**Point-based localization methods**. Most crowd counting datasets only provide point annotations rather than anchor annotations. Therefore, it is more convenient to use point annotations as the supervision information for crowd localization. Specifically, they [69,70,71,72] formulated the crowd localization problem as a foreground/background segmentation problem and used the cross-entropy loss to optimize the network.

**Heuristic-based localization methods**. The heuristic-based localization methods [17,73,74] were proposed to obtain the crowd locations from the crowd density map. In particular, they usually adopt the non-maxima suppression to obtain the maximum local value, which presents each head location in the crowd. Then, the extracted locations are matched with true head locations by 1–1 matching. The feasible solutions are obtained via the Hungary algorithm for evaluating the performance of crowd locations.

### 2.3. Remote Sensing Object Counting

Remote sensing object counting, which aims to estimate the number of ground objects from remote sensing images, is a challenging and important computer vision task. Comparing with traditional object counting in natural scenes, the task of remote sensing object counting is more challenging in several aspects: large-scale variation, extremely complex backgrounds, and orientation arbitrariness [75]. It is an important way to obtain counting information by combining classification, detection, or segmentation results in remote sensing images.

For example, Bazi et al. [76] proposed an automatic method that contained a classification step using a Gaussian process classifier (GPC) and a counting step for counting olive trees in very high spatial remote sensing images. Santoro et al. [77] proposed a four-step algorithm that consisted of an asymmetrical smoothing filter, local minimum filter, mask layer, and spatial aggregation operator for tree counting. Xue et al. [78] applied a semi-supervised method for counting mammals in the open savanna. A parallel architecture was proposed by [79] to count olive trees in a crop field, which mainly uses color-based or stereo vision-based segmentation.

In recent years, deep learning methods have dominated the remote sensing object counting task. Mubin et al. [80] proposed a deep learning framework based on LeNet to detect and count oil palm trees in remote sensing images. Shao et al. [81] proposed a detection and counting system based on Yolo V2 [82] for cattle counting. A neural network named ResCeption was proposed by [83] to count cars by regression, which combined residual learning with inception layers. Context sensing is helpful for many applications (e.g., behaviour recognition [84]), and is also important for remote sensing.

Layout Proposal Networks (LPNs) with spatial kernels were proposed to count and locate cars in drone videos, which can leverage spatial context information effectively [85]. For congested remote sensing object counting scenes, the density map-based methods are more effective than detection-based methods.

Gao et al. [86] proposed an ASPD-Net for remote sensing object counting in an encoder–decoder framework. To deal with the shortcomings of hand-crafted methods used for generating density maps, an adaptive density map generator [87] was proposed for learning a density map representation for the counter, which adopted the annotation dot information as the input. The generator and counter were trained jointly in an end-to-end manner and had good performance in remote sensing object counting.

## 3. Proposed Method

In this section, we will first introduce the problem formulation of crowd counting in this paper. Then, we describe the details of our proposed MSCA module. After that, our MSCANet and the comparisons of different context modules from the crowd counting network are presented. Finally, the details of MSCANet for extension tasks (i.e., crowd localization and remote sensing object counting) are illustrated in detail.

### 3.1. Problem Formulation

We formulate crowd counting compliance with [8,10], which considers the problem as a pixel-wise regression problem. To be specific, the density map $F_{i}$ is formed as follows:

(1) $$F_{i}\left(x\right)=\sum_{j=1}^{M}\delta \left(x-a_{j}\right)\times G_{\sigma }\left(x\right),$$

where δ(·) stands for the Dirac delta function, $G_{\sigma }$ represents the 2-D normalized Gaussian kernel, σ denotes the standard deviation, $a_{j}$ is the head location, and *M* is the total crowd number of $I_{i}$. The crowd counting network learns the non-linear mapping between the input image $I_{i}$ and its corresponding crowd density map $F_{i}$. The $L_{2}$ loss is defined as the network loss function:

(2) $$L\left(\Theta \right)=\frac{1}{2N}\sum_{i=1}^{N}{∥F\left(I_{i};\Theta \right)-F_{i}∥}_{2}^{2},$$

where Θ represents the learning parameters of MSCANet, and *N* and $F(I_{i};\Theta )$ denote the image number and the output of crowd counting network, respectively. More technically, in this paper, we introduce a new multi-scale contextual feature aggregation method, i.e., MSCA. The details are described in the next subsection.

### 3.2. Multi-Scale Context Aggregation Module

**Figure 2:**
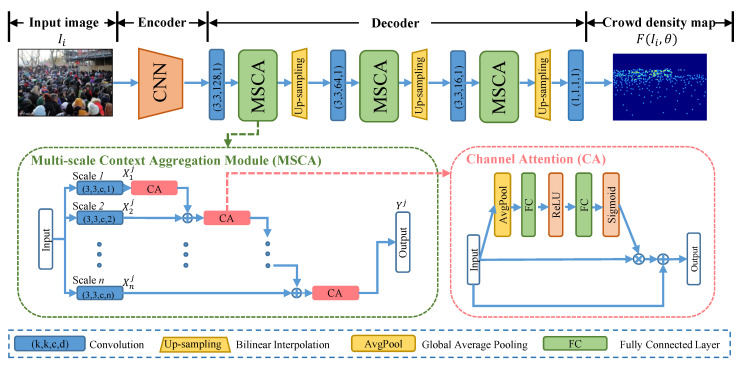
Detailedillustration of our Adaptive Multi-scale Context Aggregation Network for crowd counting.

Making full use of contextual features at different scales is an effective way to address the scale variation of people. However, small-scale context features can only represent partial cues due to the limitations of receptive fields. It is ineffective to directly aggregate the small-scale context features with large-scale context features, which will introduce irrelevant and useless cues and hinder the counting performance. Thus, we resort to a selection mechanism to adaptively select and transform typical small-scale context features for aggregating them with large-scale context features. According to this consideration, we propose a MSCA module, and its specific structure is shown in Figure 2.

The MSCA module was designed as a unified multi-branch atrous convolution layer, where each layer has a different dilated rate. Concretely, we denote *i*, *r*, and $j\;\in \;\{\frac{1}{2^{r-1}}\;\cdots \;\frac{1}{4},\frac{1}{2},1\}$ as the dilated rate, reduction ratio, and resolution of the feature map, respectively. The context feature is represented by $X_{i}^{j}\in R^{jW\times jH\times C}$. we adopt a function *f* being responsible for selecting informative features from $X_{i}^{j}$. The context features are aggregated as follows:

(3) $$Y^{j}=f\left(\cdots f\left(f\left(X_{1}^{j}\right)⊕X_{2}^{j}\right)⊕X_{3}^{j}\right)⊕\cdots ⊕X_{n}^{j}),$$

where ⊕ represents the element-wise summation and $Y^{j}\in R^{jW\times jH\times C}$ denotes the output feature of MSCA module. Specifically, We employed a channel attention(CA) [18] to instantiate the selecting function *f* without extra supervision information. As illustrated in Figure 2, the context feature is first sent to a global average pooling ($F_{avg}$) layer and subsequently processed by a bottleneck structure consisting of two fully connected layers. Finally, a sigmoid function is applied to normalize the output feature. The selecting operation not only highlights the typical features but also suppresses possible noise existing in the redundant features. The detailed process is as follows:

(4) $$\alpha_{i}=W_{2}^{fc}\left(W_{1}^{fc}\left(F_{avg}\left(X_{i}^{j}\right)\right)\right),$$

where $\alpha_{i}\in R^{jW\times jH\times C}$ denotes the adaptive coefficient. $W_{1}^{fc}$ and $W_{2}^{fc}$ represent the weights of the two fully connected layers, and the first fully connected layer is followed by a ReLU function. For better optimization, a residual connection is adopted between the input and output of CA. The residual equation is as follows:

(5) $$f\left(X_{i}^{j}\right)=X_{i}^{j}+\alpha_{i}X_{i}^{j},\;i=1\;\cdots \;n.$$

We summarize the computation process of MSCA and give its pseudocode as shown in Algorithm 1.
**Algorithm 1** Pseudocode of Multi-scale Context Aggregation Module with three branches in a PyTorch-like style.###########################       initialization       ############################branch1 = nn.Conv2d(in_channels, out_channels, kernel = 3, padding = 1, dilation = 1)branch2 = nn.Conv2d(in_channels, out_channels, kernel = 3, padding = 2, dilation = 2)branch3 = nn.Conv2d(in_channels, out_channels, kernel = 3, padding = 3, dilation = 3)avg_pool = nn.AdaptiveAvgPool2d(1)CA1 = nn.Sequential( nn.Linear(out_channels, out_channels // 4, bias = False), nn.ReLU(inplace = True), nn.Linear(out_channels // 4, out_channels, bias = False), nn.Sigmoid() )CA2 = nn.Sequential( nn.Linear(out_channels, out_channels // 4, bias = False), nn.ReLU(inplace = True), nn.Linear(out_channels // 4, out_channels, bias = False), nn.Sigmoid() )CA3 = nn.Sequential( nn.Linear(out_channels, out_channels // 4, bias = False), nn.ReLU(inplace = True), nn.Linear(out_channels // 4, out_channels, bias = False), nn.Sigmoid() )###########################       forward pass       ############################feature1 = branch1(x), feature2 = branch2(x), feature3 = branch3(x)b, c, _, _ = feature1.size()y = avg_pool(feature1).view(b, c)y = CA1(y).view(b, c, 1, 1)## Channel attention, Equation (4)channel_attention_map1 = y.expand_as(feature1)feature1 = feature1 * (1 + channel_attention_map1) ## Residual learning, Equation (5)feature2 = feature2 + feature1## Context feature aggregation, Equation (3)b, c, _, _ = feature2.size()y = avg_pool(feature2).view(b, c)y = CA2(y).view(b, c, 1, 1)## Channel attention, Equation (4)channel_attention_map2 = y.expand_as(feature2)feature2 = feature2 * (1 + channel_attention_map2)## Residual learning, Equation (5)feature3 = feature3 + feature2## Context feature aggregation, Equation (3)b, c, _, _ = feature3.size()y = avg_pool(feature3).view(b, c)y = CA3(y).view(b, c, 1, 1)## Channel attention, Equation (4)channel_attention_map3 = y.expand_as(feature3)feature3 = feature3 * (1 + channel_attention_map3)## Residual learning, Equation (5)return feature3

### 3.3. Multi-Scale Context Aggregation Network

Based on MSCA, we propose an end-to-end deep neural network, i.e., MSCANet, for congested crowd counting, which leverages context cues to effectively bootstrap the task of crowd counting and localization. The pipeline is shown in Figure 2. Given an input image $I_{i}$, we first use a CNN to encode features. Then, the encoding features are fed into multiple MSCA modules aimed to obtaining ample scale information. Specifically, we employ an up-sampling layer following each MSCA to gradually transform the multi-scale context feature map into higher-resolution representations. Finally, a convolution operation is performed on the learned multi-scale context features with a 1 × 1 convolution kernel for predicting the crowd density map.

### 3.4. Compared to Other Context Modules

**Figure 3:**
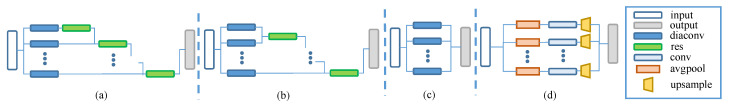
Different structures of multi-scale context modules. (**a**) Multi-scale context aggregation module (MSCA) w/o channel attention (CA); (**b**) cascade context pyramid module (CCPM); (**c**) scale pyramid module (SPM); and (**d**) scale-aware context module (SACM).

We compare MSCA with another three context modules from [15,16,88], as shown in Figure 3. To obtain a compact context feature, the Cascade Context Pyramid Module (CCPM) [88] progressively aggregates large-scale contextual representation with small-scale contextual representation, as shown in Figure 3b. The CCPM block enhances the context features as follows:

(6) $$Y^{j}=g\left(\cdots g\left(g\left(X_{n}^{j}⊕X_{n-1}^{j}\right)⊕X_{n-2}^{j}\right)⊕\cdots ⊕X_{1}^{j}\right),$$

where g(·) denotes the residual block (res) from [89]. In contrast to CCPM, we fuse contextual features from small to large in an adaptive way.

A Spatial Pyramid Module (SPM) [16] first adopts a multi-branch atrous convolution layer to encode context information. Then, the output feature of each branch is equally summated by an element-wise sum operation, as shown in Figure 3c. The learning process of SPM is as follows:

(7) $$\begin{array}{cc} Y^{j} & =\sum_{i=1}^{n}X_{i}^{j}=\sum_{i=1}^{n}W_{i}^{diaconv}\left(U\right), \end{array}$$

where $U\in R^{W\times H\times C}$ and $W_{i}^{diaconv}$ denote the input features and weights of the dilated convolution layers, respectively. Differently from SPM, rgw MSCA module adaptively selects reliable information from different scales of context information.

Liu et al. [15] employed spatial pyramid pooling [90] to capture multi-scale context features from local features, and then the contrast features were extracted from the differences between local features and multi-scale context features to enhance the representation of people at different scales. Referring to the above method, we introduce a Scale-Aware Context Module (SACM) for crowd counting as shown in Figure 3d. The SACM outputs context features as follows:

(8) $$Y^{j}=\sum_{i=1}^{n}X_{i}^{j}=\sum_{i=1}^{n}U_{p}\left(W_{i}^{conv}\left(P_{ave_{i}}\left(U\right)\right)\right),$$

where $P_{ave_{i}}\left(\cdot \right)$, $W_{i}^{conv}$, and $U_{p}$ represent the adaptive average pooling layer that averages the input feature *U* into i×i blocks and the weights of the convolution layers and bilinear interpolation operation for upsampling, respectively. Compared to SACM, we apply a different way to encode scale-aware context features. The experiments in the next section verify the superiority of our MSCA module.

### 3.5. Extension of MSCANet

We extend our MSCANet to two relevant tasks: crowd localization and remote sensing object counting. The former aims to obtain the exact locations of the crowd, and the latter aims to obtain the accurate number of remote sensing objects from remote sensing images.

**Crowd Localization**. Following [17,73,74], we also obtain the crowd localization results from the crowd density map. Specifically, we first apply our MSCANet to generate the original density map. Then, we utilize the non-maximum suppression to process the extracted crowd density map to obtain the local maximum response map, which is our final crowd localization results. The comparisons are illustrated in Section 4.3.

**Remote Sensing Object Counting**. Given that remote sensing object counting has more similarities with crowd counting, we also formulate remote sensing object counting tasks as a density estimation problem. Thus, we use the annotations from the remote sensing object counting dataset to generate a density map following Section 3.1, and directly train our MSCANet on it. The detailed comparison results will be presented in Section 4.3.

## 4. Experiments

In this section, we first introduce the datasets and implementation details. Then, we describe the evaluation metrics for crowd/remote sensing object counting and crowd localization. After that, the comparison results on test sets of different benchmarks between our MSCANet and other state-of-the-art methods for crowd counting, crowd localization, and remote sensing object counting are presented. Finally, comprehensive ablation studies were performed to evaluate the effectiveness of each component of MSCANet.

### 4.1. Datasets

We conducted comprehensive experiments on four popular datasets, i.e., ShanghaiTech_Part_A [10], UCF_CC_50 [91], UCF-QNRF [17], and RSOC [86]:

**ShanghaiTech_Part_A** [10] consists of 482 images in total (300 images for training and 182 images for testing). The crowd density varies significantly between different crowd images. Specifically, the minimum number of people is 33 while the maximum is 3139, which poses a difficult challenge for accurate estimation.

**UCF_CC_50** [91] contains 50 images, which are randomly crawled from the internet, and the maximum number of people is equal to 4543. Limited training images, and different perspectives and resolutions are challenging factors for crowd counting methods. We follow the standard setting in [91] to conduct a five-fold cross-validation.

**UCF-QNRF** [17] is a new proposed dataset, which has great improvement in the quantity and quality of crowd images. The total number of images is 1535, including 1201 training images and 334 testing images. The number of people in the UCF-QNRF dataset varies from 49 to 12,865.

**RSOC** [86] is the largest remote sensing object counting dataset, which contains 3057 images with 286,539 instances. It consists of four types of remote sensing objects, i.e., Building, Small-Vehicle, Large-Vehicle, and Ship, and the number of remote sensing object varies significantly.

We used the first ten layers of VGG-16 pre-trained on ImageNet as the backbone. The initial learning rate was 1 × 10^−5^, and the optimizer was SGD with momentum. All experiments were performed on a C^3^ Framework [92,93] with a single RTX 2080 Ti GPU card and an Intel(R) Core(TM) i7-8700 CPU with 16 GB RAM and 512 GB ROM. The experiment software environments were the Pytorch 1.1 framework, Python 3.6, CUDA 10.1, and Ubuntu 18.04 LTS operation system. The data pre-processing and augmentation strategies of the above three datasets all follow the C^3^ Framework. The training batch size was set to 4 and 1 on *UCF_CC_50* and the other datasets, respectively.

### 4.2. Evaluation Metrics

**Counting Metrics**. The mean absolute error (MAE) and mean squared error (MSE) were applied to evaluate the counting performance:

(9) $$\mathrm{MAE}=\frac{1}{N}\sum_{1}^{N}\left|z_{i}-\hat{z_{i}}\right|,$$

(10) $$\mathrm{MSE}=\sqrt{\frac{1}{N}\sum_{1}^{N}{\left(z_{i}-\hat{z_{i}}\right)}^{2}},$$

where $z_{i}$ and $\hat{z_{i}}$ denote the truth number and the predicted number of people in image $I_{i}$ respectively.

**Localization Metrics.** For the crowd localization task, we adopted the precision (P), recall (R), and F1-measure (F1) to evaluate the localization performance:

(11) $$P=\frac{TP}{TP+FP},$$

(12) $$R=\frac{TP}{TP+FN},$$

(13) $$F1=\frac{2\cdot P\cdot R}{P+R},$$

where *TP*, *FP*, and *FN* denote the number of true positive samples, false positive samples, and false negative samples, respectively. Specifically, the extracted crowd localization points were matched with ground-truth points by 1–1 matching, and the *TP*, *FP*, and *FN* were calculated under the pixel distance threshold value from 1 to 100 pixels. If the distance between the extracted point and the ground truth point was less than the pixel distance value, the localization result was marked as *TP*; if the distance between the extracted point and the ground truth point was larger than the pixel distance value, the localization result was marked as *FP*; if there existed no matched extracted point with the ground truth point, the localization result was marked as *FN*.

### 4.3. Comparison with State-of-the-Arts

#### 4.3.1. Crowd Counting

We compare our MSCANet with the top performing methods [8,9,10,17,38,39,56,70,91,94,95] on four datasets, and the comparison results are reported in Table 1.

**Performance on ShanghaiTech_Part_A.** We observed that our MSCANet achieved the best performance on MSE and competitive results on MAE compared to the other methods, which verifies the effectiveness of MSCANet. Specifically, it outperformed CSRNet by −1.7 and −12.9 in terms of MAE and MSE.

**Performance on UCF_CC_50.** Our model achieved the best performance on MAE and promising performance on MSE. More remarkably, MSCANet surpasses the performance of TEDNet [95] −6.56 and −24.68 on MAE and MSE, respectively.

**Performance on UCF-QNRF.** Our method produced the best results on both MAE and MSE and outperformed the second-best result, i.e., TEDNet [95], by −8.9 and −4.2 on the MAE and MSE metrics, respectively. The above improvements are due to the effect of MSCA, which can learn more multi-scale context features used for crowd counting.

#### 4.3.2. Crowd Localization

**Figure 4:**
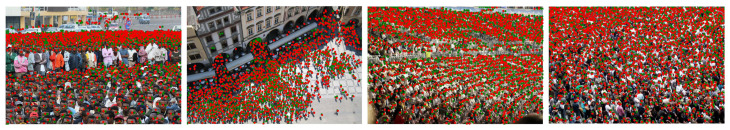
Visualizations of MSCANet for crowd localization on the UCF-QNRF dataset. Red points denote the ground-truth, and green points denote the estimated location results of MSCANet.

We conducted a crowd localization task on the UCF-QNRF dataset. The quantitative results are presented in Table 2. The performance of MSCANet outperformed the other state-of-the-art crowd localization methods in terms of the F1-measure, which demonstrates that our model can efficiently obtain the crowd localization in different crowd scenes. Figure 4 presents the crowd localization results of MSCANet. We can see that our model performed well on different crowd scenes with different crowd distributions, which further proves the effectiveness of our MSCANet.

#### 4.3.3. Remote Sensing Object Counting

**Figure 5:**
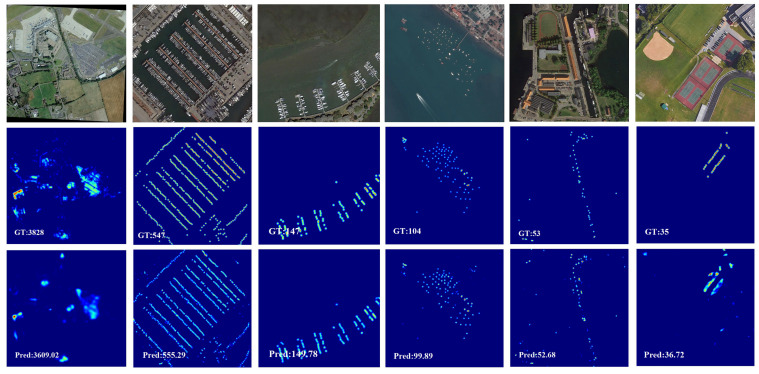
Visualization results of MSCANet for remote sensing object counting on RSOC dataset.

We perform our model on RSOC for remote sensing object counting. Table 3 displays the comparison results. We can see that our method achieves comparable results against other state-of-the-art methods. Specifically, MSCANet sets a new state-of-the-art result on *Small vehicle* and *Ship* and surpasses other state-of-the-art methods by a significant margin, which proves the effectiveness of our method. Figure 5 presents the qualitative results of our model. We find that the density map generated by MSCANet are very close to the ground truth density maps, which further prove the superiority of our model.

### 4.4. Ablation Study

#### 4.4.1. Multi-Scale Context Aggregation Module

We first evaluated the performance of MSCANet with different pyramid scale settings. The pyramid scale setting (PS) denotes what dilated convolution branches are used in MSCA module, and the value of the PS represents the dilated rate of each branch. We investigated different PS settings to determine a suitable combination. As shown in Table 4, the performance of MSCANet gradually improved as the parameter of PS increased, reaching saturation at PS = {1,2,3,}.

**Figure 6:**
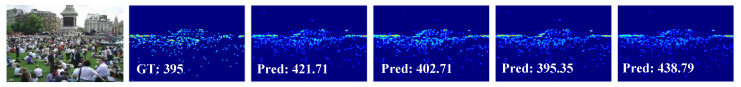
Impacts of different pyramid scale settings on UCF-QNRF. From left to right: input image, ground truth, result of PS = {1}, result of PS = {1,2}, result of PS = {1,2,3}, and result of PS = {1,2,3,4}.

Continually increasing the parameter of PS did not improve the performance of the network. This is mainly because a larger receptive field results in redundant information, which hinders the learning of multi-scale context representation. As shown in Figure 6, we visualized the output of MSCANet with different pyramid scale settings. The predicted results of PS = {1,2,3} were very close to the ground truth. Based on this analysis, we set PS = {1,2,3} in the following experiments.

**Figure 7:**
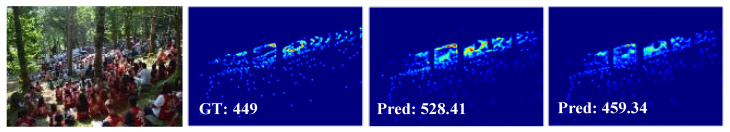
Impacts of CA on UCF-QNRF. From left to right: input image, ground-truth, result of MSCA w/o CA, and result of MSCA.

We studied the effects of the MSCA block by comparing our full model to one of the same architecture without MSCA, denoted as MSCANet w/o MSCA (Decoder). Moreover, to measure the effectiveness of CA for feature aggregation, we designed another network variant, MSCA w/o CA, by replacing the CA block with a simple residual block. Table 5 reports the comparison results of the above changes. MSCA outperformed MSCA w/o CA and Decoder in terms of MAE. The visual results in Figure 7 show the impacts of CA. We can see that MSCA w/o CA performed worse than MSCA, which further verifies the importance of CA in MSCANet.

#### 4.4.2. Multi-Scale Context Modules

We compared our MSCANet with the other prominent context-based crowd counting networks, i.e., *Congested Scene Recognition Network* (CSRNet) [8] and *Context-aware Network* (CAN) [15], which also employ the first 10 layers of VGG-16 pre-trained on ImageNet as a backbone. The detailed results are reported in Table 5. Our MSCANet achieved the top performance on both the MAE and MSE metrics.

**Figure 8:**
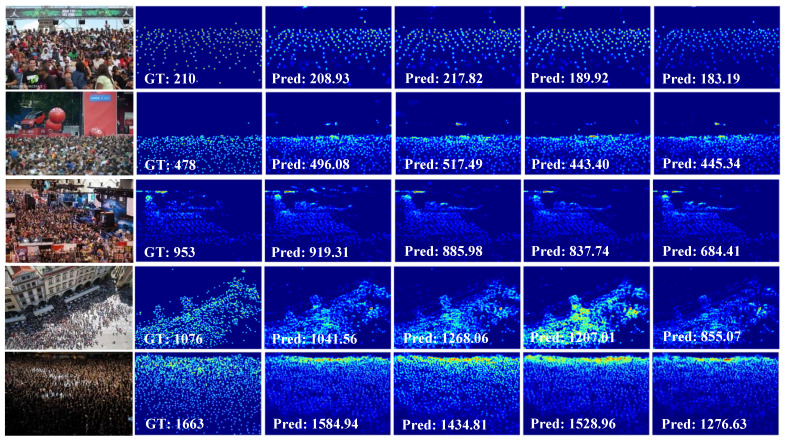
Visual comparision of different multi-scale context modules on UCF-QNRF. From left to right: input images, ground-truth, results of our method, results of CCPM, results of SPM, and results of SACM.

Then, we studied the influence of using MSCA, CCPM, SPM, and SACM. For a fair comparison, all of them had three branch structures, and the feature extractor was the same as MSCANet. The comparison results are shown in Table 5. MSCA achieved the lowest MAE on the UCF-QNRF dataset. Figure 8 displays the predicted results of typical images with different crowd density levels. The qualitative and quantitative results demonstrate that the MSCA block was critical for our model to improve performance, especially in congested scenery.

## 5. Conclusions and Future Work

In this paper, we proposed a novel MSCANet for congested crowd counting. The core of MSCANet is the MSCA block, which consists of multi-branch atrous convolution layers and channel attention modules. The atrous convolution layers aim to extract multi-scale contextual features while channel attention modules contribute to filter the redundancy features and highlight the features that are beneficial for crowd counting. Extensive experiments were performed on three congested crowd datasets, and our MSCANet achieved favorable results against the other prominent methods. Moreover, we extended our model to two relevant tasks, i.e., crowd localization and remote sensing object counting. The experimental results on UCF-QNRF and RSOC demonstrated the generalization ability of MSCANet.

However, our model only utilizes the spatial context information of a single image, and the performance of MSCANet is limited for video object counting. In future work, we will extend our model with temporal context information for the task of video object counting. Specifically, we can first count each frame using our proposed MSCANet to obtain the count result of each frame. We can obtain the global information of the video sequence from the count result of each frame. Then, with the help of the global information, we can apply the rescore method to modify the unsatisfied count result of those frames. Finally, we obtain the counting number of the video from the estimated and refined count results.

## Figures and Tables

**Table 1:** Comparison of the different state-of-the-art methods on the *ShanghaiTech_Part_A*, *UCF_CC_50*, and *UCF-QNRF* datasets.

Method	SHA		UCF_CC_50		UCF-QNRF	
	MAE	MSE	MAE	MSE	MAE	MSE
Lempitsky et al. [56]	-	-	493.4	487.1	-	-
Zhang et al. [9,10]	181.8	277.7	467.0	498.5	-	-
Idrees et al. [17,91]	-	-	419.5	541.6	315	508
MCNN, [10,17]	110.2	173.2	377.6	509.1	277	-
Switching CNN, [17,38]	90.4	135.0	318.1	439.2	228	445
CL, [17]	-	-	-	-	132	191
CP-CNN, [39]	73.6	106.4	298.8	**320.9**	-	-
CSRNet(baseline), [8]	68.2	115.0	266.1	397.5	-	-
ic-CNN(one stage), [94]	69.8	117.3	-	-	-	-
ic-CNN(two stage), [94]	68.5	116.2	-	-	-	-
CFF, [70]	65.2	109.4	-	-	-	-
TEDNet, [95]	**64.2**	109.1	249.4	354.5	113	188
**MSCANet (Ours)**	66.5	**102.1**	**242.8**	329.8	**104.1**	**183.8**

**Table 2:** Comparison of the localization results on the UCF-QNRF dataset.

Method	Av. Precision	Av. Recall	F1-Measure
MCNN [10]	59.93%	**63.50**%	61.66%
DenseNet63 [96]	70.19%	58.10%	63.87%
CL [17]	75.80%	59.75%	66.82%
SCLNet [74]	**83.99**%	57.62%	67.36%
**MSCANet (Ours)**	83.65%	61.07%	**69.64**%

**Table 3:** Comparison of the different state-of-the-art methods on *RSOC*.

Method	Building		Small Vehicle		Large Vehicle		Ship	
	MAE	MSE	MAE	MSE	MAE	MSE	MAE	MSE
MCNN [10,17]	13.65	16.56	488.65	1317.44	36.56	55.55	263.91	412.30
CMTL [97]	12.78	15.99	490.53	1321.11	61.02	78.25	251.17	403.07
CSRNet [8]	8.00	11.78	443.72	1252.22	34.10	46.42	240.01	394.81
SANet [34]	29.01	32.96	497.22	1276.66	62.78	79.65	302.37	436.91
SFCN [93]	8.94	12.87	440.70	1248.27	33.93	49.74	240.16	394.81
SPN [16]	7.74	11.48	445.16	1252.92	36.21	50.65	241.43	392.88
SCAR [98]	26.90	31.35	497.22	1276.65	62.78	79.64	302.37	436.92
CAN [15]	9.12	13.38	457.36	1260.39	34.56	49.63	282.69	423.44
SFANet [99]	8.18	11.75	435.29	1284.15	29.04	47.01	201.61	332.87
ASPDNet [86]	**7.59**	**10.66**	433.23	1238.61	**18.76**	**31.06**	193.83	318.95
**MSCANet (Ours)**	11.13	16.02	**221.16**	**430.90**	60.92	78.20	**41.93**	**60.73**

**Table 4:** Comparisons of our proposed method with different pyramid scale settings (PS) on the UCF-QNRF dataset. The value of PS is the dilated rate of each dilation convolution branch from MSCA.

PS	MAE	MSE
{1}	110.9	197.2
{1,2}	105.2	184.6
{1,2,3}	**104.1**	**183.8**
{1,2,3,4}	104.8	186.1

**Table 5:** Comparisons of our proposed method with different architecture changes on the UCF-QNRF dataset.

Configuration	MAE	MSE
Decoder (baseline)	111.3	**182.0**
MSCA w/o CA	105.7	186.9
MSCA	**104.1**	183.8
CSRNet (our reimplementation)	118.8	204.4
CAN [15]	107.0	183.0
CCPM	111.9	182.3
SPM	108.1	187.2
SACM	116.2	211.2

## Data Availability

Not applicable.
